# Targeting Coronaviral Replication and Cellular JAK2 Mediated Dominant NF-κB Activation for Comprehensive and Ultimate Inhibition of Coronaviral Activity

**DOI:** 10.1038/s41598-017-04203-9

**Published:** 2017-06-22

**Authors:** Cheng-Wei Yang, Yue-Zhi Lee, Hsing-Yu Hsu, Chuan Shih, Yu-Sheng Chao, Hwan-You Chang, Shiow-Ju Lee

**Affiliations:** 1 Institute of Biotechnology and Pharmaceutical Research, National Health Research Institutes, Miaoli, 35053 Taiwan, ROC; 2 Institute of Molecular Medicine, National Tsing Hua University, Hsinchu, 30013 Taiwan, ROC

## Abstract

Tylophorine-based compounds exert broad spectral, potent inhibition of coronaviruses. NF-κB activation is a common pro-inflammatory response of host cells to viral infection. The aims of this study were to (i) find an effective combination treatment for coronaviral infections through targeting of the virus per se and cellular NF-κB activity; and (ii) to study the underling mechanisms. We found that tylophorine-based compounds target the TGEV viral RNA and effectively inhibit TGEV replication. NF-κB inhibition also leads to anti-TGEV replication. NF-κB activation induced by TGEV infection was found to be associated with two convergent pathways, IKK-2_IκBα/p65 and JAK2 mediated p65 phosphorylation, in swine testicular cells. JAK2 inhibition either by CYT387 (a JAK family inhibitor) or by silencing JAK2-expression revealed a dominant JAK2 mediated p65 phosphorylation pathway for NF-κB activation and resulted in NF-κB inhibition, which overrode the IκBα regulation via the IKK-2. Finally, tylophorine-based compounds work cooperatively with CYT387 to impart comprehensive anti-TGEV activities. The combination treatment, wherein a tylophorine compound targets TGEV and a JAK2 inhibitor blocks the alternative dominant NF-κB activation mediated by JAK2, is more effective and comprehensive than either one alone and constitutes a feasible approach for the treatment of SARS-CoV or MERS-CoV.

## Introduction

Coronaviruses are animal viruses containing an enveloped, positive-sense, single-stranded RNA genome; and include the common cold human coronavirus (CoV)-229E & CoV-OC43, severe acute respiratory syndrome (SARS) CoV, Middle East respiratory syndrome (MERS) CoV, porcine transmissible gastroenteritis virus (TGEV), and murine hepatitis virus (MHV) etc^1–3^. Since the 2003 SARS outbreak (which had a mortality rate of ~10%), novel anti-SARS-CoV treatments have been vigorously pursued. No new cases of SARS have been reported since the 2003 outbreak, but another novel coronavirus (MERS-CoV), this time with a mortality rate of ~35%, came to light in 2012^4^. To date, neither a commercially available vaccine for human coronaviruses nor a specific treatment for SARS-CoV or MERS-CoV is available.

Coronaviruses primarily infect the upper respiratory and gastrointestinal tract and induce host inflammation^5^. Nuclear factor κB (NF-κB) activation by coronaviruses is usually responsible for mediating the production of pro-inflammatory cytokines and chemokines and thus plays an important role in the pathogenesis of disease caused by coronaviruses^6, 7^. Inhibition of NF-κB-mediated inflammation induced by coronavirus in mice was reported to increase the survival rate^6^. Thus, NF-κB inhibitors constitute a promising class of antivirals in infections caused by pathogenic coronaviruses. However, targeting NF-κB is limited by intrinsic pathway complexity, cross talk with other pathways, and drug resistance^8^. In addition, many small chemical molecules targeting either viral entry or the intracellular viral life cycle were reported to impart anti-coronavirus activity^2, 9, 10^. Despite these advances, however, SARS-CoV and MERS-CoV remain untreatable diseases for which novel therapies are sought.

Tylophorine based compounds, whether isolated from plants, e.g. Asclepiadaceae and Moraceae or chemically synthesized, exert potent anti-coronaviral activities against a variety of coronaviruses including SARS-CoV, MHV, and TGEV^11, 12^, but both the underlying mechanism(s) of this inhibitory effect and the target are unknown. NF-κB is significantly activated after infection by coronaviruses, e.g. TGEV, SARS-CoV, MERS-CoV, and MHV etc^6, 7, 13–15^. Otherwise, coronaviral nucleocapsid (N) protein plays a vital role in the regulation of viral genome replication and host gene transcription^16^. Therefore, the combination treatment for the inhibition of coronavirus per se, e.g. viral genome replication, and blocking cellular NF-κB activation by coronaviruses, is a promising approach for the development of anti-coronavirals.

TGEV infected swine testicular (ST) cells constitute a surrogate system for the search and study of potential anti-coronavirus agents^11, 12, 17^. Herein, we report: (1) tylophorine-based compounds exert potent anti-TGEV replication by directly targeting the viral RNA/ribonucleoprotein (RNP) complex for viral replication/synthesis; (2) NF-κB inhibition also leads to anti-TGEV replication; (3) A combination treatment consisting of both a tylophorine compound and an NF-κB inhibitor acts additively or synergically to more effectively and comprehensively inhibit TGEV replication than either (1) or (2) alone.

## Results

### Tylophorine-based compounds interacted with TGEV viral RNA/RNP and inhibited TGEV RNA replication

**Figure 1:**
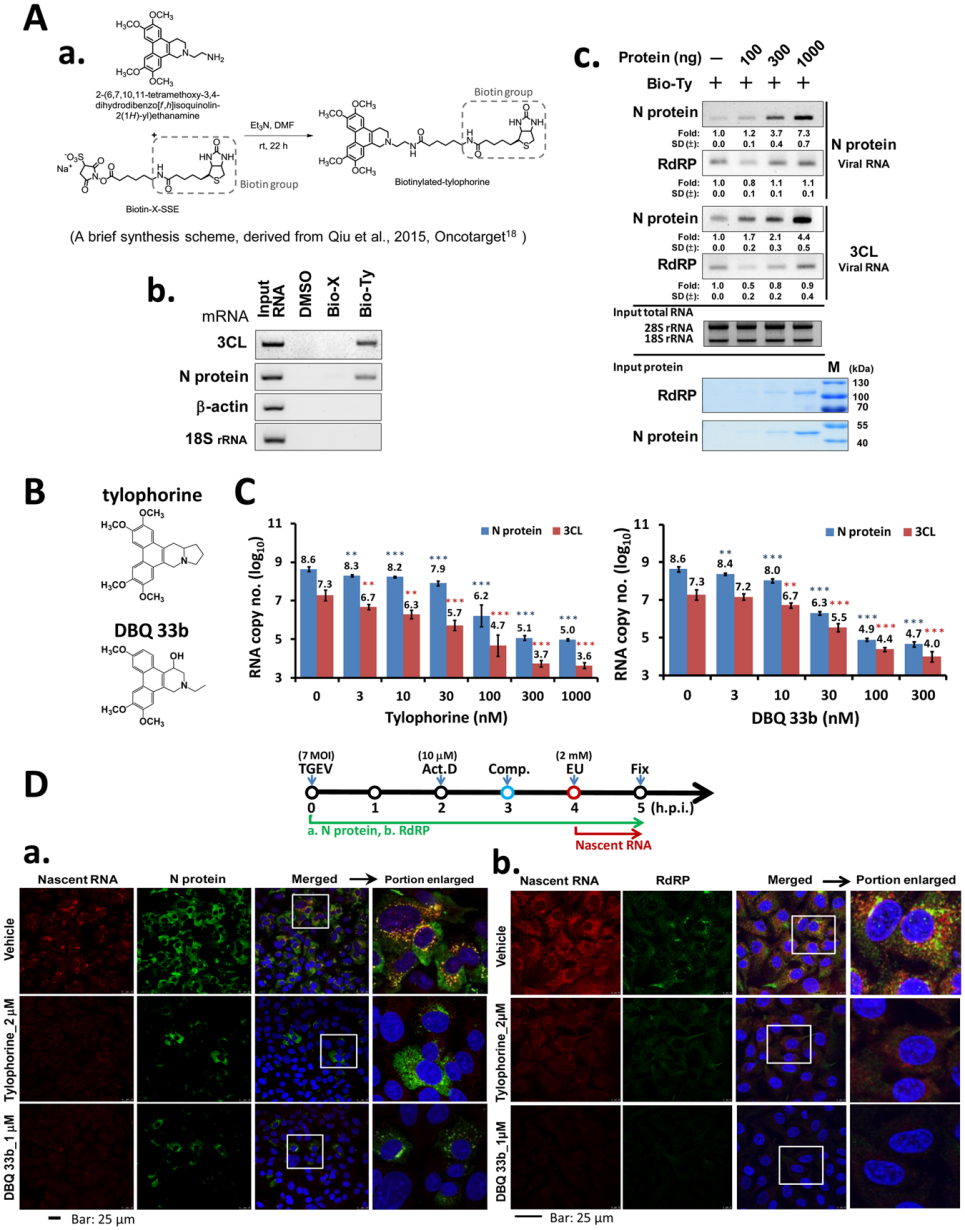
Tylophorine compounds inhibited TGEV RNA replication through interaction with TGEV viral RNA/RNP. (**A**) Tylophorine compound interacted with TGEV viral RNA. (a) A brief synthesis scheme, derived from Qiu *et al*.^18^. (b) Biotinylated tylophorine interacted with TGEV viral RNA. (c) TGEV N protein enhanced the association of biotinylated tylophorine with TGEV viral RNA. The total RNAs (5 µg) extracted from TGEV infected ST cells at 6 h.p.i. were subjected to pull-down experiments with 30 µM of biotinylated tylophorine or Biotin-X-SSE at 4 °C for 4 h in the absence or presence of recombinant N protein or His-RdRP with the indicated amount. The pulled down complexes were subjected for analysis by RT-PCR with TGEV specific primers, designed from the regions of TGEV N or 3CL^pro^ gene body. 18/28S ribosomal RNAs were used as the input controls for pull-down assays. Biotinylated tylophorine and Biotin-X-SSE were respectively labeled as Bio-Ty and Bio-X. (**B**) Chemical structures of tylophorine and DBQ 33b. (**C**) Tylophorine compounds inhibited TGEV replication. TGEV infected ST cells at an MOI of 7, treated with the indicated doses of compounds at 6 h.p.i. were harvested and subjected for total RNA extraction and the determination of the viral copy numbers (see Materials and Methods). Shown are means ± S.D. from three independent experiments each in triplicates. (**p < 0.01 & ***p < 0.001; versus no compound treatment). (**D**) Tylophorine compounds inhibited TGEV nascent viral RNA replication and N and RdRP protein expression. TGEV infection, compound treatments and EU addition for labeling nascent RNA were performed in the sequence in the upper scheme as the shown black arrow line. The green arrow line represented the period of N (a) or RdRP (b) protein expression since TGEV infection and the red one for the nascent RNA synthesis since EU addition. Actinomycin D (Act. D) was added at 2 h.p.i. to inhibit DNA-dependent RNA polymerase. For immunofluorescent staining, following fixation, the cells were detected for the incorporated alkyne-modified EU with azide-derivatized Alexa 594 fluorophores by using click chemistry for nascent viral RNA (red), immuno-stained with FITC-mouse IgG for anti-TGEV N (a) or RdRP (b) protein (green) and stained with 4′,6-diamidino-2-phenylindole (blue) for visualizing nuclei. The fluorescence image was acquired by using a Leica TSC SP5 laser-scanning confocal microscope. Bar: 25 μm. Uncropped images for Fig. 1A are presented in Supplementary Figure S7.

Tylophorine compounds exert potent anti-coronavirus activities^11, 12^, but the mechanism of action remains unknown. Previously, we reported that tylophorine-based compounds exert anti-cancer activity predominantly by targeting the RNP complex containing caprin-1 protein/c-Myc mRNA in carcinoma cells^18^. Accordingly, we investigated whether the observed anti-TGEV activity arises from the targeting of viral RNP. First, we demonstrated that biotinylated tylophorine, not Biotin-X-SSE (see Fig. 1A-a for chemical structures), interacted with purified TGEV viral RNAs in a pull-down experiment using streptavidin beads (see materials and methods) (Fig. 1A-b). This interaction of biotinylated tylophorine and TGEV viral RNA was enhanced by addition of recombinant TGEV N protein, but not RNA-dependent RNA polymerase (RdRP), in a dose dependent manner (Fig. 1A-c). Moreover, a cellular colocalization of TGEV viral nascent RNA, N protein and fluorescent tylophorine compound surrounding the nuclei in TGEV infected cells was also observed (Figs S1 and S2). Secondly, through RT-qPCR, we also demonstrated tylophorine based compounds (Fig. 1B) profoundly inhibited TGEV viral replication by diminishing viral RNA copies by up to ~4 orders of magnitude (Fig. 1C). Thirdly, using 5-ethynyl uridine (EU) labelled newly synthesized RNA, we clearly demonstrated that tylophorine compounds, either tylophorine or DBQ 33b, inhibited the nascent viral RNA synthesis and thus blocked the colocalization of viral nascent RNA with either N protein (Fig. 1D-a) or RdRP (Fig. 1D-b) around nuclei.

### NF-κB activation in TGEV infected ST cells was associated with IKK-2_IκBα/p65 axis and JAK pathway mediated p65 phosphorylation

**Figure 2:**
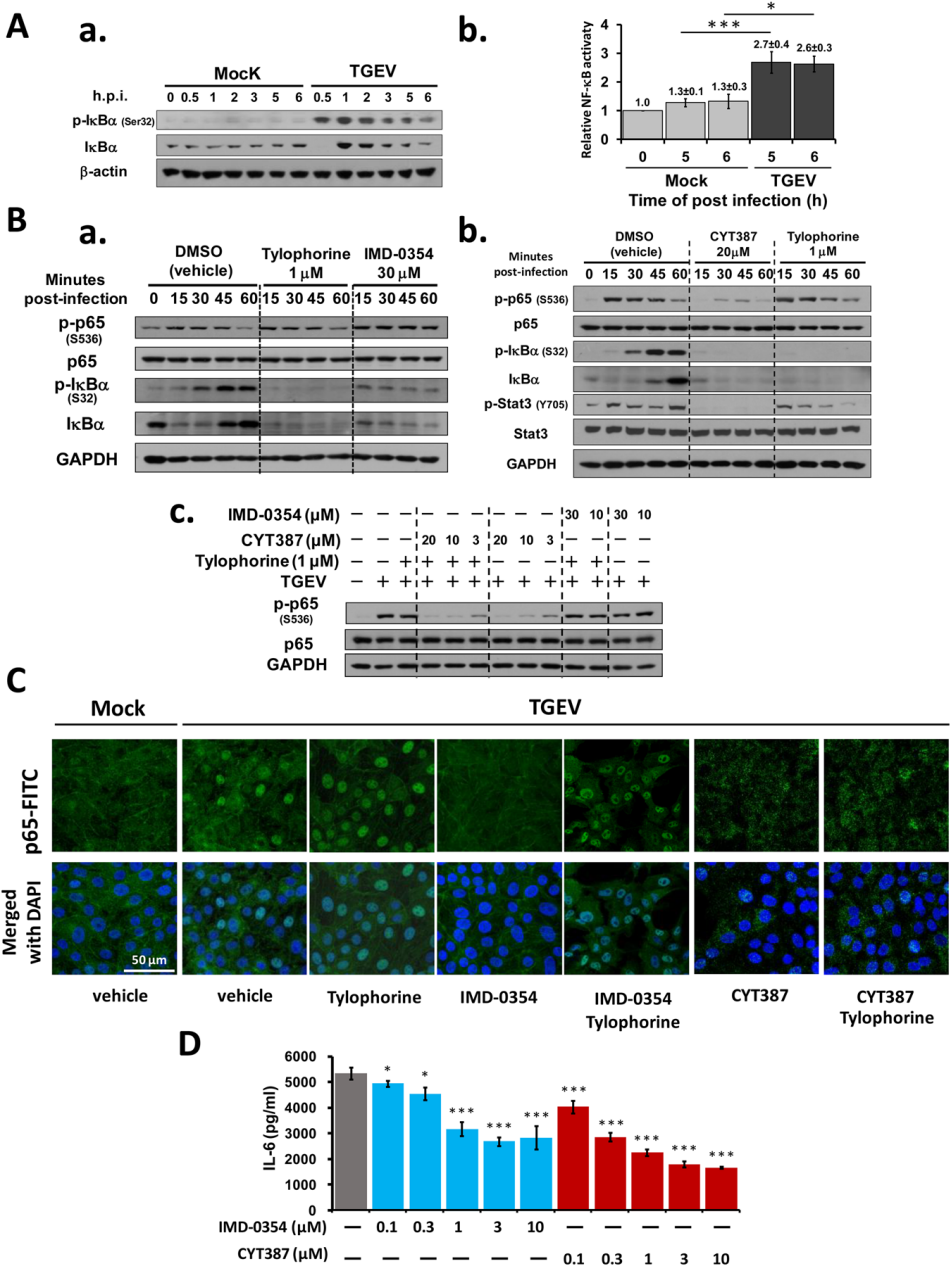
NF-κB activation in TGEV infected ST cells was associated with IKK-2_IκBα/p65 axis and JAK pathway mediated p65 phosphorylation. (**A**) NF-κB activation in TGEV infected ST cells. (a) TGEV infection induced IκBα protein expression and phosphorylation. Cells, either infected with TGEV or not, were harvested at the indicated h.p.i. and subjected to western analysis with the antibodies indicated. (b) NF-κB activation was induced by TGEV infection. An NF-κB–luciferase reporter was used to monitor the levels of NF-κB activation (See Materials and Methods). Shown are the representative of three independent experiments (a) or means ± S.D. from three independent experiments, each performed in triplicate (b). (*p < 0.05 & ***p < 0.001). (**B**) Differential inhibition on the phosphorylation of IκBα and p65 by IMD-0354, CYT387 and tylophorine respectively. (a) IMD-0354 inhibited IκBα phosphorylation and tylophorine diminished the IκBα protein level. The phosphorylated p65 was not decreased by IMD-0354 or tylophorine. (b) JAK inhibitor CYT387 inhibited p65 phosphorylation and diminished the IκBα protein level in TGEV infected ST cells. (c) The combined treatment of tylophorine with CYT387, but not with IMD-0354, inhibited p65 phosphorylation in TGEV infected ST cells. Cells were treated with vehicle DMSO, tylophorine, IMD-0354, CYT387, or the indicated combination for 1 h prior to TGEV infection and harvested at the indicated minute post-infection (m.p.i.) (for a & b) or 15 m.p.i. (for c) for western analyses with the antibodies indicated. (**C**) The effects of IMD-0354, CYT387 and tylophorine on p65 translocation into nucleus. IFA assays were performed for TGEV infected ST cells at 45 m.p.i. with the indicated compound treatment. All the cells were treated with vehicle DMSO, tylophorine (1 µM), IMD-0354 (30 µM), CYT387 (20 µM) or the combination of tylophorine with either IMD-0354 or CYT387 for 2 h prior to TGEV infection. Results shown are representative of three independent experiments. Bar: 50 μm. (**D**) IL-6 induction was inhibited either by CYT387 or IMD-0354 treatment. Supernatants of TGEV infected ST cells (7 MOI) with various treatments at 6 h.p.i. were harvested respectively to determine the amounts of IL-6 secreted by ELISA kits. (*p < 0.05 & ***p < 0.001; versus no compound treatment) Uncropped images for Fig. 2A and B are presented in Supplementary Figure S8.

Virus-induced NF-κB activation is involved in cytokine induction, and associated with inflammation^19^. NF-κB was activated in ST cells upon TGEV infection, as demonstrated by a western analysis for phosphorylation in IκBα (Fig. 2A-a) and a luciferase reporter assay for NF-κB activation (Fig. 2A-b). IMD-0354, an IKK-2/NF-κB inhibitor, was applied to diminish the phosphorylation on IκBα and p65. IMD-0354 profoundly inhibited IκBα phosphorylation (Fig. 2B-a) and decreased the amounts of phosphorylated p65 translocated into nuclei (Figs 2C and S3), thereby conceivably inhibiting NF-κB activation. However, a significant amount of phosphorylated p65 persisted in TGEV infected ST cells in the treatment with IMD-0354 (Fig. 2B-a). Therefore, it was reasoned that other pathway(s) exists for NF-κB activation in the TGEV infected ST cells, which are capable of mediating p65 phosphorylation even when the IKK-2_IκBα_p65 axis is inhibited by IMD-0354.

After a search of pathway pharmacological inhibitors for the additional effector pathways that mediate the phosphorylation of p65, we found CYT387, a JAK pathway inhibitor, diminished phosphorylation of p65 in TGEV infected ST cells (Fig. 2B-b) even in the presence of tylophorine (Fig. 2B-c). Moreover, CYT387 diminished the protein level of IκBα and its phosphorylated form (Fig. 2B-b), as did tylophorine. However, tylophorine moderately increased the levels of p65 in TGEV infected ST cells’ nuclei (Fig. 2C). Finally, CYT387, but not IMD-0354, was able to significantly diminish the p65 phosphorylation in the presence or absence of tylophorine in TGEV infected cells, as demonstrated in western analysis (Fig. 2B-c) and immunofluorescence assay (IFA) for p65 translocation to nuclei (Fig. 2C). As expected, IMD-0354 and CYT387 individually and significantly blocked the induction of IL-6, a NF-κB target gene, in TGEV infected ST cells (Fig. 2D).

### Differential combinatory effects of tylophorine with NF-κB inhibitors; Tylophorine worked in an additive manner with CYT387 but antagonized IMD-0354 for the TGEV inhibition

**Figure 3:**
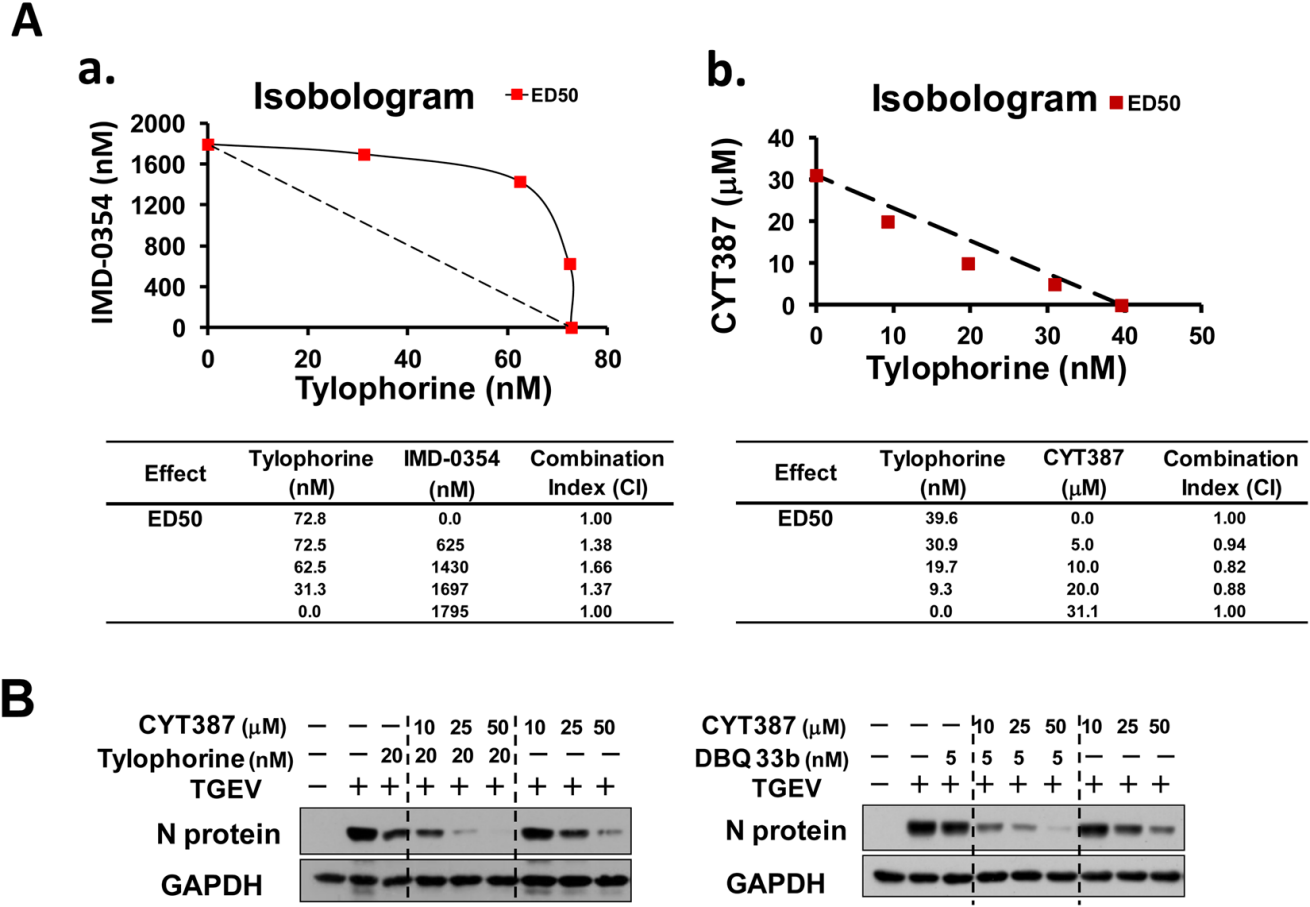
Differential combinatory effects of tylophorine with NF-κB inhibitors. (**A**) Tylophorine antagonized IMD-0354 but worked in an additive manner with CYT387 for the TGEV inhibition. (a) Tylophorine and IMD-0354 exerted antagonistic effect in inhibiting TGEV replication. (b) Tylophorine and JAK inhibitor CYT387 inhibited TGEV activity by an additive manner. All the ST cells were treated with vehicle DMSO, tylophorine, IMD-0354, CYT387, or the combination of tylophorine with IMD-0354 or CYT387 for 1 h prior to TGEV infection and detected by immunofluorescence staining at 6 h.p.i. Drug combination tests were monitored by IFA as described in Materials and Methods. TGEV infected ST cells showed a poor adherence property in the presence of CYT387 in 96-well culture plates but exhibited a much better adherence property in 24-well culture plates. Therefore, the combination tests for tylophorine and IMD-0354 were performed in 96-well culture plates and for tylophorine and CYT387 in 24-well culture plates. Isobologram plots of effective dose ED_50_ are shown at upper panels. The combination index (CI) values at ED_50_ were calculated using Calcusyn software and summarized in the tables at lower panels. CI = 1 refers to additive effect, CI < 1 refers to synergism, and CI > 1 means antagonism. ED_50_ represents effective doses of 50% inhibition. Results shown are representative of three independent experiments. (**B**) CYT387 facilitated anti-TGEV activity of tylophorine (or DBQ 33b) by further diminishing TGEV N protein expression. Mock or TGEV transfected ST cells were treated with vehicle DMSO, tylophorine, DBQ 33b, or CYT387 as indicated concentrations for 1 h prior to TGEV infection and harvested for western analyses at 6 h.p.i. with the indicated antibodies for N protein and GAPDH. Results shown are representative of three independent experiments. Uncropped images for Fig. 3B are presented in Supplementary Figure S9.

Since tylophorine, IMD-0354, and CYT387 (a JAK family inhibitor) exhibited differential effects on the regulation of IκBα and p65 in TGEV infected ST cells, drug combination experiments were performed to investigate their combined effects on anti-TGEV replication using the IFA assays described in Materials and Methods. IMD-0354 was able to exert anti-TGEV activities with an EC_50_ (50% maximal effective concentration) of 1.8 ± 0.1 µM. The combination index values for ED_50_ (50% maximal effective dose) of tylophorine and IMD-0354 were calculated using Calcusyn software and all were in the range of ~1.3 to ~1.7. Thus, tylophorine and IMD-0354 both inhibited TGEV activity, but in an antagonistic manner (Fig. 3A-a). In contrast, CYT387 was able to exert anti-TGEV activity with an EC_50_ of 30.4 ± 1.2 µM; CYT387 and tylophorine worked additively in anti-TGEV activity with the combination index values at ~0.8–0.9 for ED_50_ (Fig. 3A-b). Lastly, CYT387 treatment was able to enhance the anti-TGEV activity of tylophorine or DBQ 33b in a dose dependent manner as determined by N protein expression (Fig. 3B), consistent with the additive effect of the combined treatment, CYT387 and tylophorine, for anti-TGEV activity (Fig. 3A-b).

### Combined treatments of tylophorine-based compounds with CYT387 cooperatively resulted in a comprehensive and ultimate reduction in TGEV titers

**Figure 4:**
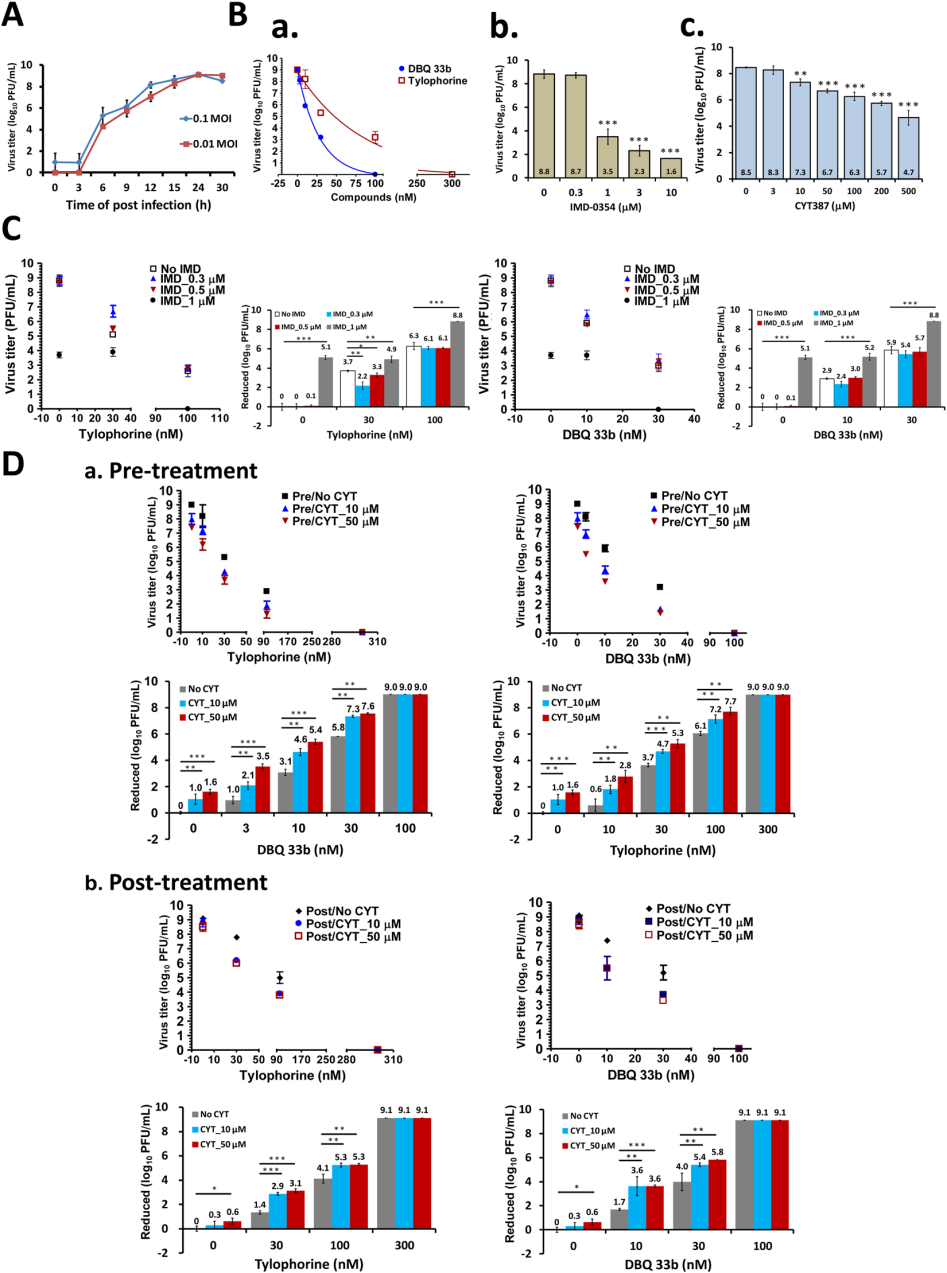
Effects of tylophorine-based compounds, IMD-354, or CYT387 alone or in combination on reducing TGEV yields. (**A**) The kinetics of TGEV replication in ST cells at MOI of 0.1 and 0.01. (**B**) Dose dependent effects of tylophorine compounds, IMD-354, and CYT387 on reducing TGEV yields. (**p < 0.01 & ***p < 0.001; versus no compound treatment). (**C**) Combined treatments of tylophorine-based compounds with IMD-354 resulted in an antagonistic reduction in TGEV yields. (*p < 0.05). (**D**) Combined treatments of tylophorine-based compounds with CYT387 resulted in a cooperatively comprehensive reduction in TGEV yields either in pre-treatment (a) or post-treatment experiments (b). (*p < 0.05 & **p < 0.01 & ***p < 0.001) ST cells were seeded the day before compound treatment or TGEV infection. The tested compounds were added to the wells 1 h prior to (**B**,**C**, **D**–a) or after (**D**–b) the addition of TGEV at an MOI of 0.01. The resultant cultures were then incubated for an additional 3–30 h (**A**) or 15 h (**B**,**C**,**D**) at 37 °C as indicated. The supernatant of cells in each specific treatment was collected and subjected to viral titer determination via an end-point dilution assay conducted with ST cells. Results shown were means ± S.D. from three or more independent experiments.

After infection in ST cells, TGEV exhibited a log phase increase in viral yields within the first ~12 h and reached a plateau from 15 to 24 h.p.i. with titers of ~10^9^ PFU (plague forming unit)/ml (Fig. 4A). Treatments with tylophorine, DBQ 33b, IMD-0354, or CYT387 all significantly blocked viral replication and diminished the viral yields in TGEV infected ST cells in a dose dependant manner (Fig. 4B). Treatment with either tylophorine or DBQ 33b completely blocked viral replication and reduced viral titers at the concentration of 300 nM or 100 nM respectively, resulting in a reduction of viral yield by 9 orders of magnitude. IMD-0354 decreased the viral yield from 10^8.8^ to 10^1.6^ PFU/ml, 7.2 orders of magnitude, at the concentration of 10 µM. CYT387, though limited by its poor solubility and moderate cytotoxicity (Fig. S4), achieved a 3.8 order of magnitude decrease in viral yield (10^8.5^ to 10^4.7^ PFU/ml) at a concentration of 500 µM. Both arms of inhibition, either through targeting TGEV viral complex or cellular NF-κB activation, were able to profoundly reduce viral replication.

When ST cells were treated with a combination of IMD-0354 and either tylophorine (30 nM or 100 nM) or DBQ 33b (10 nM or 30 nM), viral titers were reduced in an antagonistic manner; that is to say, reduction in titers achieved by the combined treatment was less than the sum of viral titer reductions achieved by the IMD-0354 and tylophorine (or DBQ 33b) separately (Fig. 4C). These results are consistent with the aforementioned antagonistic effect (Fig. 3A).

On the other hand, when ST cells were treated with a combination of CYT387 with either tylophorine or DBQ 33b, the opposite effect – an additive or synergistic effect – was observed (Fig. 4D). For example, when 10 µM or 50 µM of CYT387 (lower than the IC_50_ value of CYT387 for anti-TGEV activity) was combined with tylophorine (10 nM, 30 nM or 100 nM) or DBQ 33b (3 nM, 10 nM or 30 nM), all the combined treatments exerted an additive manner or a synergistic effect for reduction of TGEV viral titers. The maximum synergic effect was about one order of magnitude (Fig. 4D). Thus, the reduction of viral yield by CYT387 was cooperatively enhanced when combined with tylophorine or DBQ 33b (Fig. 4D-a). These results are consistent with the aforementioned additive effect (Fig. 3) for tylophorine based compounds and CYT387. Moreover, post-treatments resulted in a more enhanced reduction of viral titers than pre-treatment (Fig. 4D-b).

### Tylophorine blocked *de novo* protein synthesis of IκBα through arresting the RNP containing caprin-1 protein and IκBα mRNA: a mechanism account of tylophorine antagonizing IMD-0354 for anti-TGEV activity

**Figure 5:**
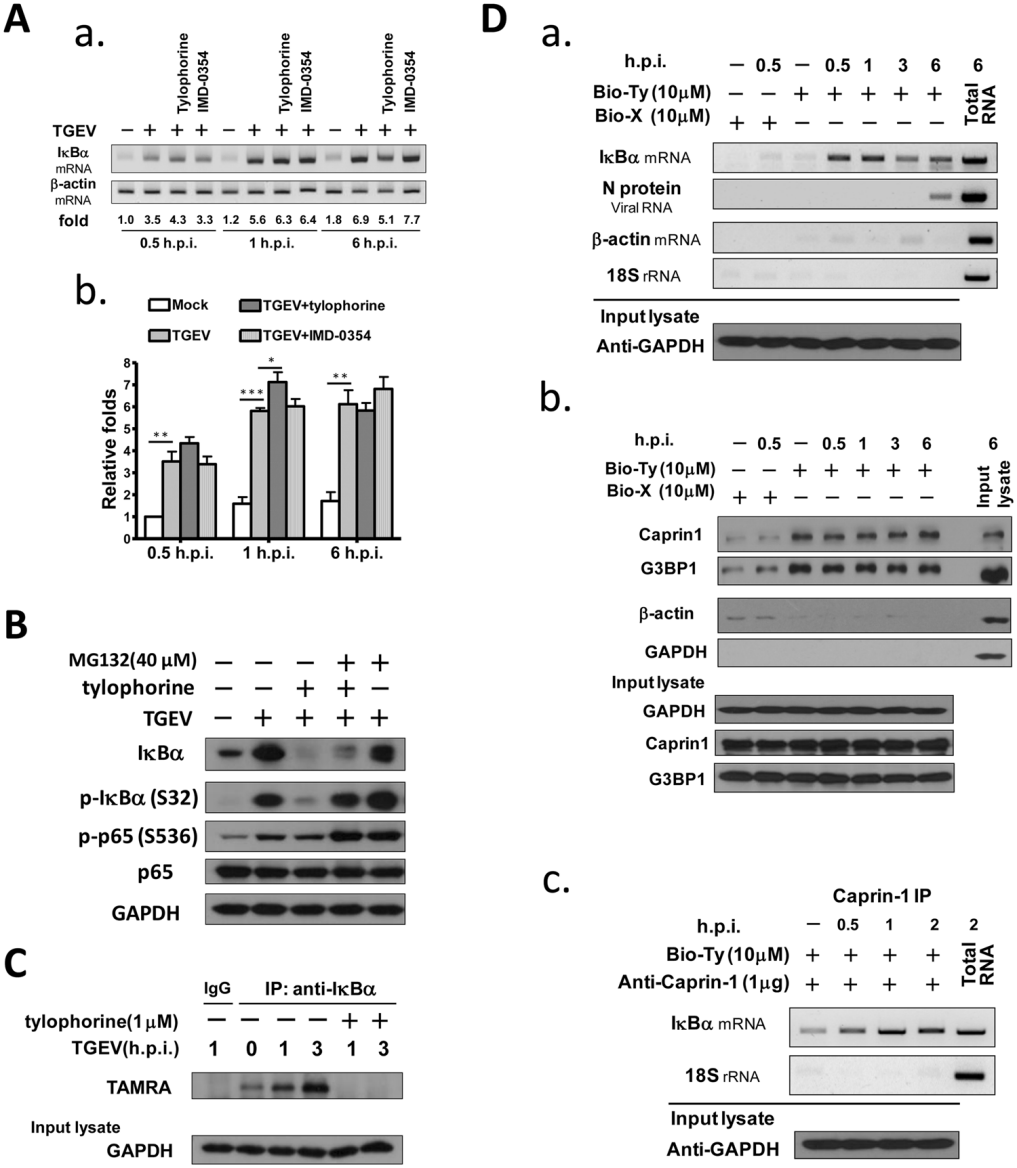
Tylophorine suppressed *de novo* protein synthesis of IκBα by arresting IκBα mRNA associated ribonucleioprotein complex for translational inhibition. (**A**) Effect of tylophorine on the induction of IκBα mRNA levels. ST cells were pretreated with tylophorine (1 µM) or IMD-0354 (30 µM) for 1 h prior to TGEV infection with the time period indicated. The relative levels of IκBα mRNA were normalized with the control β-actin. (**B**) Tylophorine diminished IκBα protein levels which were independent of proteolysis. ST cells were pretreated with MG132 for 1 h and then followed by tylophorine treatment for another 1 h prior to TGEV infection. Cells were harvested at 1 h.p.i. of TGEV infection and the resultant lysates subjected to western analysis with the antibodies indicated. (**C**) Tylophorine inhibited IκBα *de novo* protein synthesis. The TGEV infected ST cells, co-cultured with Click-iT^®^ HPG, were harvested and subjected to immunoprecipitation by an anti-IκBα followed by western analysis with an antibody against TAMRA for newly synthesized IκBα. (**D**) Tylophorine was associated with the RNP complex of IκBα mRNA and caprin-1 protein. Biotinylated tylophorine interacted with IκBα mRNA (a) and capriin-1 protein (b). IκBα mRNA was co-immunoprecipitated with anti-caprin-1 (c). ST cells were infected with TGEV and harvested at the indicated h.p.i. for preparation of lysates for pull-down experiments (a and b). The resultant total lysates (600 µg) were incubated with 10 µM of biotinylated tylophorine or Biotin-X-SSE for 4 h and the pulled down mRNA complexes, after TRIzol extraction, were subjected to RT-PCR with the indicated specific primer pairs (a) or the pulled down proteins were subjected to western analysis with the indicated antibodies for caprin-1 and G3BP1 (b). Or, the infected ST lysates were incubated with an antibody against caprin-1 in the presence of biotinylated tylophorine and the immunoprecipitates were subjected for mRNA isolation and subsequent RT-PCR for analysis of relative IκBα mRNA or 18 S rRNA levels (c). Biotinylated tylophorine: Bio-Ty; Biotin-X-SSE: Bio-X. Results shown are representative of three independent experiments or means ± S.D. of three independent experiments. (*p < 0.05 & **p < 0.01 & ***p < 0.001) Uncropped images for Fig. 5 are presented in Supplementary Figure S10.

To elucidate how IκBα protein expression was eliminated by tylophorine (Fig. 2B), we first examined whether tylophorine downregulates the transcription of IκBα and thus inhibits IκBα protein expression by RT-PCR for detecting the amounts of IκBα mRNA in TGEV infected cells treated with tylophorine or IMD-0354. IκBα mRNA was significantly induced upon TGEV infection in ST cells. However, tylophorine did not decrease the TGEV induced IκBα mRNA levels as manifested at 0.5, 1, and 6 h.p.i. (Fig. 5A). Thus tylophorine did not diminish IκBα protein level by transcriptional downregulation. Secondly, we examined whether proteasomal degradation was involved. Tylophorine disabled the induction of IκBα and its phosphorylated form at 1 h.p.i by TGEV. MG132, a proteasome inhibitor, was able to significantly increase the level of the phosphorylated IκBα upon TGEV infection. However, MG132, when co-administered with tylophorine, was not able to recover IκBα protein level (after its elimination by tylophorine), whereas the level of phosphorylated IκBα form was recovered to the level induced by TGEV. This is attributed to the fact that phosphorylated IκBα is the very form subjected to proteasome for degradation and MG132 functions to block this process (Fig. 5B). Thus, tylophorine did not undergo blocking proteasomal degradation to reduce IκBα protein level. Thirdly, we examined whether tylophorine blocked *de novo* protein synthesis of IκBα protein using a Click-iT^®^ HPG and TAMRA labelling/detecting method to trace the newly synthesized protein^18, 20^. As shown in Fig. 5C, the amount of newly synthesized IκBα significantly increased with time after TGEV infection. Treatment with tylophorine completely eliminated the *de novo* synthesis of IκBα (Fig. 5C).

Since tylophorine was reported to block c-Myc protein expression by arresting the RNP complex containing caprin-1 protein and c-Myc mRNA in c-Myc highly expressed carcinoma cells^18^, we further examined whether tylophorine also blocked IκBα protein expression, which was induced upon TGEV infection (Fig. 2A-a), through a similar mechanism. A biotinylated tylophorine compound was applied to the pull-down experiments with cell lysates and subsequently western analyses or RNA extraction for RT-qPCR were used to identify the interacting molecules, e.g. IκBα mRNA or caprin-1. IκBα mRNA was pulled down by biotinylated tylophorine but not Biotin-X-SSE from TGEV infected ST cells. IκBα mRNA was also not pulled down in the mock transfected cells (Fig. 5D-a). Caprin-1 protein was pulled down in significant amounts by biotinylated tylophorine but not Biotin-X-SSE from lysates of mock or TGEV infected ST cells within the first 6 h.p.i., as was G3BP1 protein, a component of caprin-1 associated RNP complex^18, 21^ (Fig. 5D-b). Furthermore, IκBα mRNA, not 18S rRNA, was co-immunoprecipitated by anti-caprin-1, in the presence of biotinylated tylophorine in TGEV infected ST cells (Fig. 5D-c).

Therefore, we suggest that tylophorine interacts with and arrests the RNP complex of caprin-1 protein and IκBα mRNA, thereby blocking IκBα protein expression induced by TGEV infection. Tylophorine reduced the IκBα protein level, and consequently p65 sequestered by IκBα was liberated and subjected to phosphorylation and NF-κB activation in TGEV infected ST cells. IMD-0354 inhibited IKK-2 thereby inhibiting both phosphorylation of IκBα and NF-κB activation. However, a CYT387 associated pathway other than IKK-2 mediated an alternative and dominant p65 phosphorylation and which also led to NF-κB activation. Therefore, tylophorine antagonized IMD-0354 mediated anti-TGEV activity through liberating p65 via diminishing the IκBα protein expression.

### CYT387 blocked JAK2-mediated p65 phosphorylation in TGEV infected ST cells; a mechanistic explanation for the additive effect of tylophorine and CYT387 for anti-TGEV activity

**Figure 6:**
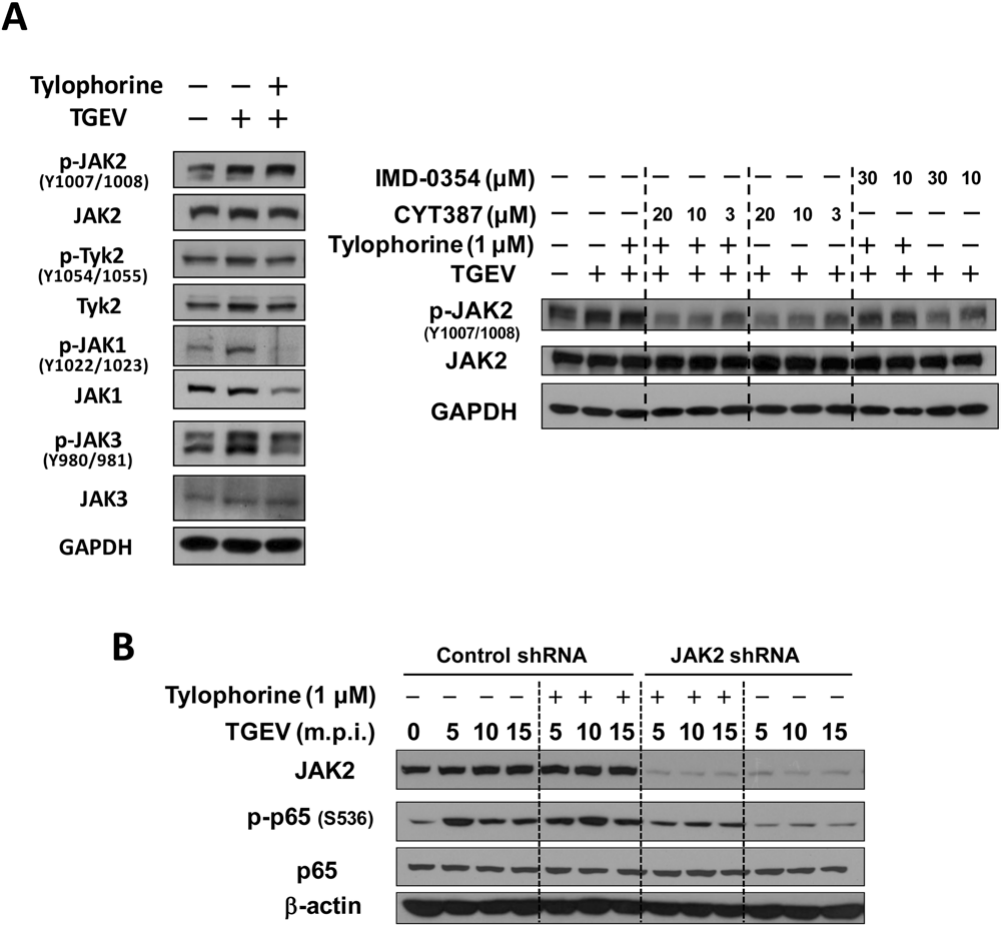
JAK2 mediated an alternative dominant p65 phosphorylation in TGEV infected ST cells. (**A**) CYT387 diminished JAK2 phosphorylation in the presence and absence of tylophorine in TGEV infected ST cells. Cells were treated with vehicle DMSO, tylophorine, IMD-0354, CYT387, or the combination of tylophorine and CYT387 for 1 h prior to TGEV infection and harvested at 15 m.p.i. for western analyses with the indicated antibodies for JAK1, p-JAK1, JAK2, p-JAK2, JAK3, p-JAK3, Tyk2, p-Tyk2, and GAPDH. The concentrations of IMD-0354 or CYT387 (µM) were used as indicated and 1 µM of for tylophorine was used. (**B**) JAK2 knockdown diminished the phosphorylation of p65 in TGEV infected ST cells. Control shRNA or shJAK2 were transduced to ST cells to knockdown JAK2 expression. The resultant JAK2-knockdown ST cells or ST cells harbored control shRNA were infected by TGEV respectively at an MOI of 7 in presence or absence of 1 µM tylophorine. Cells were harvested at 5, 10 and 15 m.p.i. of TGEV and the resultant cell lysates were subjected to western analysis with the indicated antibodies for JAK2, p65, p-p65 (S536) and β-actin. Uncropped images for Fig. 6 are presented in Supplementary Figure S11.

The identity of the specific member of the JAK family responsible for inhibiting p65 phosphorylation by CYT387 was sought. JAK1, JAK2, JAK3, and Tyk2 all became more phosphorylated after infection of ST cells by TGEV. Moreover, these increased phosphorylation events of JAK1, Tyk2, and JAK3, except that of JAK2, were decreased by tylophorine treatment (Fig. 6A left panel). Furthermore, the phosphorylation events of JAK2 in the presence or absence of tylophorine were all inhibited by CYT387 in a dose dependent manner (Fig. 6A right panel).

The effect of JAK2 knockdown on the phosphorylation of p65 in TGEV infected ST cells was also studied. Control shRNA or shJAK2 were transduced to ST cells for a vector control and knockdown JAK2 expression. The resultant JAK2-knockdown ST cells or ST cells harbored control shRNA were infected by TGEV respectively in the presence or absence of tylophorine (Fig. 6B). The results showed that: (1) the p65 phosphorylation was dramatically diminished in the JAK2-knockdown ST cells; and (2) in the presence of tylophorine treatment, p65 phosphorylation was significantly decreased in JAK2 knockdown cells compared to that with control shRNA. Accordingly, we concluded that JAK2 mediated an alternative but dominant p65 phosphorylation in the TGEV infected ST cells and JAK2 inhibition by CYT387 or knockdown by gene silencing was able to profoundly block the p65 phosphorylation with or without tylophorine treatment.

**Figure 7:**
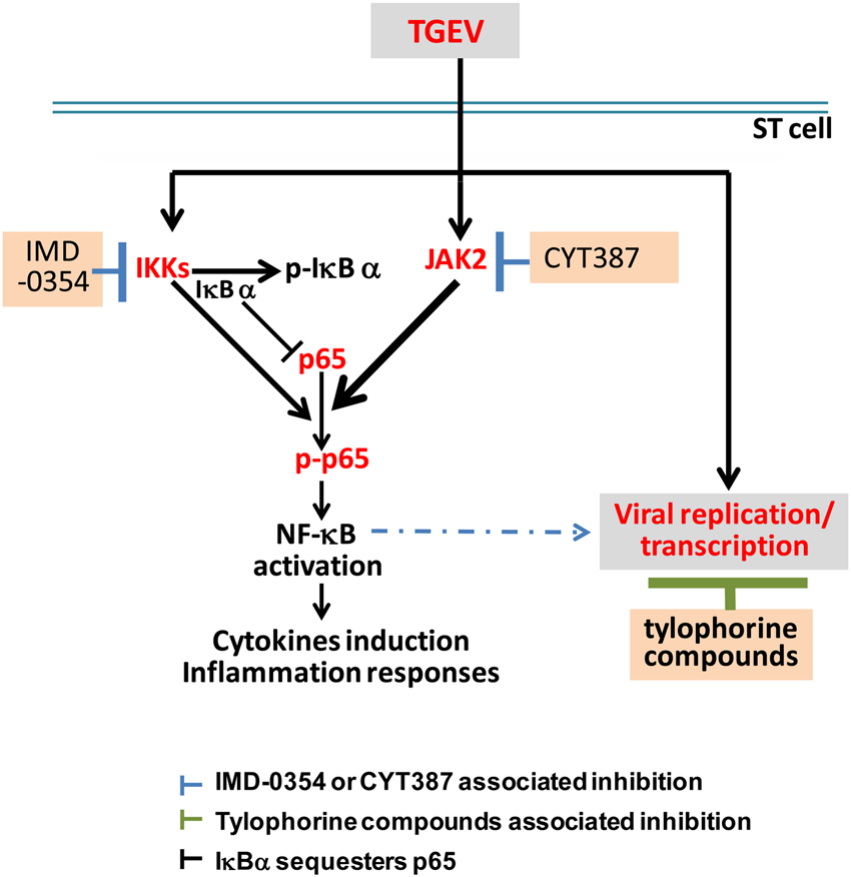
An illustrative scheme for effective points of tylophorine compounds, IMD-0354, and JAK inhibitor CYT387 in TGEV infected ST cells.

Since tylophorine arrested IκBα mRNA, reduced IκBα protein expression, and liberated p65 (Figs 2B,C and 5), the diminishment of p65 phosphorylation via JAK2 inhibition by CYT387 (Figs 2B-c and 6A) was reasoned to be capable of working in an additive manner with tylophorine for anti-TGEV activity (Figs 3 and 4). Thus, in addition to the high potency of anticoronaviral tylophorine, simultaneously blocking the viral induced NF-κB from host cells could diminish the occurrence of inflammatory response, e.g. IL-6 (Fig. 2D), and therefore constitute an effective, comprehensive and cooperative anti-coronaviral strategy (Fig. 7).

## Discussion

Human coronaviruses constitute a grave threat to public health, a situation exemplified by the outbreaks of SARS-CoV in 2003 and MERS-CoV in 2012. To date, however, there is still no specific antiviral treatment or vaccine available for human CoV infection^2, 22^, and the current standard of care for affected individuals focuses on symptom relief and, in severe cases, support for vital organ function^2, 22^.

Cytokine storms and other inflammation factors in persons infected with SARS or MERS are usually the result of NF-κB activation^6, 14^. Accordingly, targeting NF-κB to effect symptom relief and help the host combat CoV replication is a sensible approach, especially when combined with antiviral compounds. Tylophorine compounds exert potent, orally active, broad spectrum, anti-coronaviral activity with EC_50_ values in the low nM ranges for SARS-CoV, MHV, and TGEV^11, 12^. Herein, TGEV was used as a surrogate system for coronavirus to dissect the underlying mechanism of action for anticoronaviral tylophorine compounds. In TGEV infected ST cells, tylophorine compounds target N protein associated viral replication complex (Fig. 1) and CYT387 blocks JAK2 mediated NF-κB activation (Figs 2 and 6), respectively. We found that the combination of tylophorine with CYT387 was a good strategy for the elimination of TGEV in ST cells and this combination strategy opens a promising future avenue for research into treatments for SARS-CoV or MERS-CoV.

While the interaction of biotinylated tylophorine and TGEV viral RNA was enhanced by the addition of recombinant TGEV N protein (Fig. 1A), the recombinant TGEV N protein exerted no effect on the interaction of biotinylated tylophorine and IκBα mRNA (Fig. S5). Therefore, future development of tylophorine derivatives that retain the binding character for TGEV viral RNP complex but not caprin-1/IκBα mRNA RNP complex is thought to be worthwhile. These selective tylophorine derivatives will provide more options for combined treatments with NF-κB inhibitors.

Moreover, TGEV N protein was reported to bind the viral genomic and subgenomic TRS and facilitate viral transcription and replication^23, 24^. The degree of RNA occupancy by chaperone proteins (e.g. N protein) delicately regulates the biological properties and functions of the RNA. The structural switch of a nucleoprotein complex from a crowded state to an overcrowded one (e.g overloaded N protein) will result in the loss of RNA function (e.g. freeze the viral transcription/replication)^25, 26^. Tylophorine binding may increase the N protein loading to or also prevent the release of N protein from the replication RNP complex. Therefore, it is suggested that tylophorine binds to and freezes the function of TGEV N protein/viral RNA complex, thereby disabling viral replication and transcription. More detailed investigations are needed to support this mode of interaction. Collectively, targeting of the viral RNA replication complex endows tylophorine compounds with the high potency for anticoronaviral activity, and reduces the likelihood of resistance.

Multiple signalling pathways or networks might cooperate or orchestrate NF-κB activation in a biological system^27^. Antiviral agents may have differential pharmacological effects in NF-κB activation. Therefore, the combined treatment of antiviral agents with NF-κB inhibitors should avoid antagonistic effects. Interestingly, in TGEV infected ST cells, IMD-0354 significantly inhibited the phosphorylation of IκBα but not that of p65 (Fig. 2B), indicating other existing pathway(s) that alternatively mediate p65 phosphorylation independent of IKK-2 mediated pathway. Obviously, there are at least two key pathways associated with NF-κB activation in the TGEV infected ST cells: one through IKK-2_IκBα_p65 phosphorylation; and the other mediated by JAK2_p65 phosphorylation (Figs 2, 3 and 5).

Regarding the character of tylophorine in arresting IκBα mRNA and blocking IκBα protein expression (Fig. 5), it can be reasoned that IMD-0354, which inhibits the pathway of IKK-2_IκBα, cannot synergize with tylophorine to exert anti-TGEV activity in ST cells. Nonetheless, blocking the JAK2 mediated p65 phosphorylation/NF-κB activation pathway efficiently diminished the p65 phosphorylation and nuclear translocation by CYT387 treatment in tylophorine co-treated, TGEV infected ST cells (Fig. 2). JAK2 knockdown by gene silencing further bolstered the dominant role of JAK2 in p65 phosphorylation/NF-κB activation in TGEV infected ST cells. Phosphorylation of p65 was dramatically decreased in TGEV infected JAK2 deficient cells compared to that in shRNA control cells with or without tylophorine treatment (Fig. 6B). Collectively, tylophorine antagonized inhibition of IKK-2_IκBα/p65 axis by IMD-0354 but worked in an additive manner with inhibition of JAK pathway mediated p65 phosphorylation by CYT387 for the TGEV inhibition. Other JAK inhibitors that confer JAK2 inhibition in TGEV infected cells also have the potential to work with tylophorine compounds to block JAK2 mediated p65 phosphorylation for further applications (Fig. S6).

The interactions between host, pathogen and anti-viral agent are complicated, and the anti-viral effect of a single agent is easily compromised. Thus, an optimal combined therapy should constitute a more effective anti-coronaviral. In addition to the high potency of anticoronaviral tylophorine, simultaneously blocking the viral induced inflammatory response from host cells could diminish the occurrence of cytokine storms, e.g. IL-6. Thus the combination treatment via targeting N protein associated coronaviral RNA synthesis by tylophorine compounds and blocking JAK2 mediated NF-κB activation by CYT387 offers an effective and ultimately cooperative anticoronaviral strategy (Fig. 7).

In conclusion, tylophorine compounds directly target viral RNA complex and completely block viral replication and reduce viral titers. We also found that JAK2 inhibition by CYT387 not only inhibited NF-κB activation (p65 phosphorylation) and subsequent cytokine production, e.g. IL-6, but also worked cooperatively with tylophorine to deliver a comprehensive and ultimate anti-TGEV activity.

## Methods

### Cells, viruses, and IFA for measurement of anti-TGEV activity and the end point dilution assay for determining virus titers by compound treatments

ST cells and the Taiwan field-isolated virulent strain of TGEV were grown and propagated as described^17^. IFA was performed as described^17^. Briefly, ST cells in 96-well (7 × 10^4^ cells/well) or 24-well (3 × 10^5^ cells/well) plates, with or without a 1 h pre-treatment with either tylophorine or the indicated inhibitors, were infected with TGEV at a multiplicity of infection (MOI) of 7. IFA was performed 6 h post-infection (h.p.i.) with antibodies against the spike (S) and N proteins of TGEV. The cells were treated with 8–10 different concentrations of test compounds. The results of these assays were used to obtain the dose–response curves from which EC_50_ values were determined.

An end point dilution assay for determining virus titers was performed as described^28^. Briefly, the supernatant obtained from the culture of TGEV infected ST cells (MOI of 0.1 or 0.01) with the indicated treatment was subjected to viral titer determination via the end point dilution assay and the Reed Muench method was used to determined TCID50 (Tissue Culture Infective Dose).

### Chemical reagents

Dimethylsulfoxide (DMSO) was used as a vehicle control for drug treatments. Chemicals and reagents were purchased from the following sources: high-performance liquid chromatography-grade DMSO from Sigma–Aldrich (St Louis, MO); Biotin-X-SSE from Invitrogen (Carlsbad, CA, USA); IMD-0354 and MG132 from Merck Millipore Calbiochem (Billerica, MA); CYT387 from Selleck Chemicals (Houston, TX, USA); FuGENE^®^ 6 transfection reagent from Promega (Madison, WI, USA); pNF-κB-Luc from Stratagene (La Jolla, CA, USA); 5-Ethynyl Uridine (EU) from Molecular Probes, Inc.

### Biotinylated-tylophorine pull-down assay

The purified total RNAs (5 µg) or cell lysates (600 µg) of TGEV infected ST cells (MOI of 7) harvested at indicated h.p.i. were incubated with Biotin-X-SSE or biotinylated tylophorine, 30 µM for RNAs and 10 µM for lysates respectively, at 4 °C for 4 h. The associated complexes were then pulled down using streptavidin Dynabeads M280 (Invitrogen) at 4 °C for 1.5 h and then subjected to western analysis or RNA isolation for RT-PCR. Western blot was used to identify the protein components from the pull-down. The biotinylated tylophorine-bound RNAs were eluted from the aforementioned pull-down with TRIzol Reagent (Invitrogen) and RT-PCR was further used to analyze the associated components.

### Plasmid construction and purification of TGEV N recombinant protein and TGEV RdRP (RNA-dependent RNA polymerase) His-tagged recombinant protein (His-RdRP)

A plasmid encoding the GST-N fusion protein, pGEX-6P-1-PEN, was constructed by inserting a TGEV N coding region into the GST-expressing vector pGEX-6P-1 (GE Healthcare). *Escherichia coli* BL21(DE3) which harbors the pGEX-6P-1-PEN was cultured at 37 °C overnight. The overnight bacterial culture was diluted with culture medium to OD600 = 0.5 and 1 mM isopropyl-thio-β-D-galactoside (IPTG) was added to induce GST-N protein expression at 30 °C for 4 h. The resultant bacterial pellets were subjected to GST-N protein purification using glutathione–sepharose beads (Sigma–Aldrich) per manufacturer recommendations. N protein was obtained from GST-N digested with PreScission Protease by following manufacturer’s instructions (GE Healthcare, Buckinghamshire, UK). Another plasmid encoding the His-RdRP fusion protein, pET28a(+)-RdRP, was constructed by inserting a TGEV RdRP coding region into the His-expressing vector pET28a(+) (Novagene). *Escherichia coli* Rosetta^TM^ (DE3)pLysS which harbors the pET28a(+)-RdRP was cultured at 37 °C overnight. The overnight bacterial culture was diluted with culture medium to OD600 = 0.5 and 1 mM IPTG was added to induce His-RdRP protein expression at 25 °C for 5 h. The resultant bacterial pellets were subjected to His-RdRP protein purification using Ni Sepharose 6 Fast Flow resin (GE Healthcare) per manufacturer recommendations. The purified proteins, TGEV N or His-RdRP, were analyzed by SDS-PAGE and quantified by a BCA protein assay (Bio-Rad).

### Generation of monoclonal antibodies against TGEV RdRP

The antigen used to generate the monoclonal antibodies was a synthesized peptide containing residues 93 to 124 (VAEHDFFTYKERFCEFGNVARRNLTKYTMMDL) derived from TGEV RdRP protein. Mice were immunized with 0.1 mg aforementioned TGEV RdRP peptide conjugated to 0.1 mg ovalbumin carrier protein. Generation of monoclonal antibodies against TGEV RdRP was contracted out to LTK Laboratories (Taiwan, R.O.C.) and carried by manufacturer’s protocols.

### Confocal microscopy and immunofluorescence staining

Immunofluorescence staining was performed as previously described^29^. For the immunofluorescence staining of p65, the antibody against p65 (Cell Signaling) and fluorescein isothiocyanate (FITC)-conjugated anti-mouse immunoglobulin (Capell Inc.) were used. The immunofluorescence staining of TGEV N or RdRP protein and EU incorporated *de novo* RNA was performed as follows. The Click-iT^®^ RNA Alexa Fluor^®^ 594 (Imaging Kit^®^ from Invitrogen) was applied to label EU for detection of nascent viral RNA in TGEV infected ST cells, per manufacturer recommendations. In general, ST cells grown on coverslips (8 × 10^5^ cells/well; 12-well plate) were infected with TGEV at an MOI of 7, and subsequently treated with 10 µM actinomycin D to inhibit DNA dependent RNA polymerase at 2 h.p.i. and tylophorine or DBQ 33b at 3 h.p.i. Then, EU (2 mM) was added at 4 h.p.i. at 37 °C and incubated for another hour to allow the incorporation of EU into newly synthesized RNAs. The resultant cells were fixed using 4% paraformaldehyde and permeabilized using 0.25% Triton X-100 at 5 h.p.i. Subsequently, the fixed cells were incubated with Alexa 594-azide solution for 30 min at RT in the dark, which ligates EU to couple the Alexa 594 red fluorescent moiety for later detection, and washed three times with PBS. Subsequently, cells were incubated with primary monoclonal antibody for TGEV N^17^ or RdRP protein at 4 °C. After three to five washes, the cells were added to solutions of FITC-conjugated anti-mouse immunoglobulin (Capell Inc.) for N or RdRP protein, and then incubated for another 1 h at RT. Subsequently, nuclei were stained with 4′,6-diamidino-2-phenylindole (DAPI) for 15 min at RT. After three to five washes, the resultant coverslips were mounted using VECTASHIELD^®^ Mounting Medium (Vector Laboratories, Burlingame, CA). The fluorescence imaging was acquired by using a Leica TSC SP5 laser-scanning confocal microscope as described previously^29^.

### Western blot analysis

Western blotting was performed as described^17, 29^. The cell lysates or pulled down complexes were subjected to SDS-polyacrylamide gel electrophoresis for western blotting analyses with antibodies against β-actin (Millipore Merck Chemicon, Pittsburgh, PA); G3BP1 (Santa Cruz); p65, p-p65 (S536), IκBα, p-IBα (S32), p-IKKα/β (S180/181), p-Stat3 (Y705), Stat3, JAK1 (Y1022/1023), JAK1, p-JAK2 (Y1007/1008), JAK2, p-JAK3 (Y980/981), JAK3, Tyk2, p-Tyk2 (Y1054/Y1055), glyceraldehyde 3-phosphate dehydrogenase (GAPDH) (Cell Signaling Technology); Caprin-1 (Proteintech Group) and horseradish peroxidase-conjugated secondary antibodies (PerkinElmer). Enhanced chemiluminescence detection reagents (Western Blot Chemiluminescence Reagent Plus; PerkinElmer) were used according to the manufacturers’ instructions to detect antigen–antibody complexes.

### Transfection and luciferase assay

ST cells were seeded at a density of 2 × 10^5^ cells/well in 24-well plates and grown in the culture medium. Six hours later, cells were transfected with pNF-κB-Luc reporter plasmids (10 ng/well) using FuGENE6^®^ according to manufacturer’s recommendation. After incubation for 18 h, cells were infected with TGEV at an MOI of 7. The mock or infected cells were harvested at 0, 5 and 6 h.p.i. for luciferase assays. Subsequently, the medium was removed, 150 µl of Glo lysis buffer (Promega) was added per well, and the resultant lysates were subject to luciferase activity analysis. Luciferase activity was normalized to the protein concentration of each cell lysate.

### RNA isolation and RT-qPCR

These experiments were performed as described^17^. Mock or TGEV infected (MOI of 7) lysates were processed and prepared at the indicated time points with or without compound treatment, and total RNA was extracted with TRIzol reagent (Invitrogen). The relative mRNA expression levels were determined by semi-RT-qPCR, analyzed with the Gel-Pro Analyzer program (Media Cybernetics), and normalized with the housekeeping gene β-actin when needed. RNA complexes from pull-down experiments, either by biotinylated tylophorine/Biotin-X-SSE or immunoprecipitated by anti-caprin antibody, were subjected to TRIzol extraction prior to RT-qPCR analyses. To quantify the viral copy numbers in the indicated RT-qPCR analyses, known amounts (numbers) of plasmids, pGEX6p-1-PEN (see above Materials & Methods) and pGEX6p-TGEV-3CL^pro^ ^17^, were used to set up the standard curves for calculation of viral copy numbers in each treatment. The primer pairs used for RT-qPCR were: 5′-CCAAGGATGGTGCCATGAAC-3′ and 5′-GGACTGTTGCCTGCCTCTAGA-3′ for N protein; 5′-ATGAAGGATGTCCTGGCAGTGT-3′ and 5′-ACCACCGTACATTTCTCCTTCAAA-3′ for 3CL^pro^; 5′-CACCAACTACAATGGCCACACA-3′ and 5′-TAGCCCTGGTAGGTGACTCTGTT-3′ for IκBα; and 5′-GGCTCAGAGCAAGAGAGGTATCC-3′ and 5′-GGTCTCAAACATGATCTGAGTCATCT-3′ for β-actin; and 5′-CCCGTCGGCATGTATTAGCT-3′ and 5′-TGTCCCACCGTCTCCTACCT-3′ for 18S ribosomal RNA.

### IL-6 cytokine measurement

The supernatant of TGEV infected ST cells (MOI of 7) with an indicated treatment at 6 h.p.i. was harvested and diluted to proper concentrations to determine the amount of IL-6 secreted using enzyme-linked immunosorbent assay kit for porcine IL-6 (R&D Systems, Inc.), per the manufacturer’s recommendations.

### TAMRA labeling for detection of *de novo* synthesized proteins

This assay was performed as described^18, 20^. Briefly, ST cells were washed with PBS and cultured with methionine-free DMEM (GIBCO-Life Technologies) at 37 °C for 1 h prior to the addition of 1 μM tylophorine and 50 μM Click-iT^®^ HPG (L-homopropargylglycine [a reactive methionine analog], Invitrogen) for another 1 h and followed by infection of TGEV at an MOI of 7 for 1 or 3 h. The cells were then harvested and subjected to the ‘click’ reaction with 20 µM TAMRA (tetramethylrhodamine, Invitrogen) for labelling the *de novo* synthesized proteins in Click-iT® Protein Reaction Buffer according to the manufacturer’s protocol (Invitrogen). The resultant cell lysates were immunoprecipitated with anti-IκBα (Cell Signaling Technology) overnight at 4 °C with constant agitation prior to incubation with protein G agarose (Millipore) at 4 °C for another 2 h. After three to five washes, the specific immunoprecipitated protein was eluted and analyzed by western immunoblot analysis with the antibody against TAMRA (Thermo Fisher Scientific).

### JAK2 gene silencing

ST cells were seeded in culture plates at 50% confluence a day prior to transduction with pseudotyped lentivirus containing human JAK2 shRNA (clone ID: TRCN0000003179) or negative control shRNA (shLacZ, clone ID: TRCN72224)(Taiwan and National RNAi Core Facility, Academia Sinica, Taiwan). At 24 h post transduction, the cells were cultured in the presence of 2 µg/ml puromycin for selection.

### Statistical Analysis

The statistical significance between two groups was evaluated by 2-tailed unpaired Student’s t test and *, **, and *** were used to denote the statistical significance for p < 0.05, p < 0.01, and p < 0.001 respectively.

## Electronic supplementary material

Supplementary Information_data set

Supplementary Information
