# Supplementary material for: Targeting Coronaviral Replication and Cellular JAK2 Mediated Dominant NF-κB Activation for Comprehensive and Ultimate Inhibition of Coronaviral Activity
Source: Sci Rep. 2017 Jun 22;7:4105. doi: 10.1038/s41598-017-04203-9 (PMC5481340; doi:10.1038/s41598-017-04203-9)
Supplement: Supplementary file 1 — Supplementary Information_data set [file 41598_2017_4203_MOESM1_ESM.doc]

**Targeting Coronaviral Replication and Cellular JAK2 Mediated Dominant**

**NF-κB Activation for Comprehensive and Ultimate Inhibition of Coronaviral Activity**

Cheng-Wei Yang^1^, Yue-Zhi Lee^1^, Hsing-Yu Hsu^1^, Chuan Shih^1^, Yu-Sheng Chao^1^, Hwan-You Chang^2^, Shiow-Ju Lee^1^,*

**Supplementary figure S7**

**Figure1 A b.**

**Figure1 A c.**

**Supplementary figure S8**

**Supplementary figure S9**

**Supplementary figure S10**

**Supplementary figure S11**
