# Supplementary material for: Targeting Coronaviral Replication and Cellular JAK2 Mediated Dominant NF-κB Activation for Comprehensive and Ultimate Inhibition of Coronaviral Activity
Source: Sci Rep. 2017 Jun 22;7:4105. doi: 10.1038/s41598-017-04203-9 (PMC5481340; doi:10.1038/s41598-017-04203-9)
Supplement: Supplementary file 2 — Supplementary Information [file 41598_2017_4203_MOESM2_ESM.doc]

**Supplementary Information**

**Targeting Coronaviral Replication and Cellular JAK2 Mediated Dominant**

**NF-κB Activation for Comprehensive and Ultimate Inhibition of Coronaviral Activity**

Cheng-Wei Yang^1^, Yue-Zhi Lee^1^, Hsing-Yu Hsu^1^, Chuan Shih^1^, Yu-Sheng Chao^1^, Hwan-You Chang^2^,

Shiow-Ju Lee^1^,*

^1^ Institute of Biotechnology and Pharmaceutical Research, National Health Research Institutes, Miaoli 35053

^2^ Institute of Molecular Medicine, National Tsing Hua University, Hsinchu 30013, Taiwan

*** Corresponding author**

**Email:** [**slee@nhri.org.tw**](mailto:slee@nhri.org.tw)

**Contents:**

**Figure S1.** Synthesis and NMR data of fluorescein conjugated tylophorine (FCT). ----------p.2

**Figure S2.** Colocalization of TGEV viral nascent RNA, N protein and fluorescent tylophorine compound in TGEV infected cells.----------------------------------------p.3

**Figure S3.** Tylophorine treatment increased the p-p65 and p65 levels in nucleus fraction from

TGEV infected ST cells. --------------------------------------------------p.4

**Figure S4.** Cytotoxicity assays for tylophorine, CYT387, and IMD-0354 in ST cells.---------p.5

**Figure S5.** Association of biotinylated tylophorine with IκBα mRNA were independent

of TGEV N protein.-------------------------------------------------------------------------p.6

**Figure S6.** Effects of JAK inhibitor AT9283 on the phosphorylation of JAK2 and p65. ----p.7

**Reference.-**------------------------------------------------------------------------------------------------p.7

**Figure S7-S11 for uncropped images** --------------------------------See Supplementary data set

**A.**

**B.**

**Figure S1. Synthesis and NMR data of fluorescein conjugated tylophorine (FCT)**. **A**. Shown are the scheme for synthesis of FCT, with a purity of 93.87% analyzed by reverse phase-HPLC. Compound 1 was prepared as previously described^1^ and coupled to BODIPY-FLC5-SE using Et_3_N in DMF at room temperature for 22 h to produce compound **2,** FCT. **B.** NMR data of FCT, compound **2**: Red crystals; ^1^H-NMR (300 MHz, CDCl_3_): 1.75 (4H, bs), 2.19 (3H, s), 2.29 (2H, bs), 2.47 (3H, s), 2.92 (2H, bs), 3.07 (2H, bs), 3.19 (2H, bs), 3.28 (2H, bs), 3.66 (2H, bs), 4.03 (6H, s), 4.10 (6H, s), 4.27 (2H, bs), 6.02 (1H, s), 6.20 (1H, d, J=3.9 Hz), 6.75 (1H, d, J=3.9 Hz), 6.95 (1H, s), 7.03 (1H, s), 7.22 (1H, s), 7.77 (1H, s), 7.78 (1H, s). ESI-MS m/z 699 (M + H)+.

**Figure S2. Colocalization of TGEV viral nascent RNA, N protein and fluorescent tylophorine compound in TGEV infected cells.** TGEV infection, actinomycin D (Act. D), tylophorine conjugated fluorescein (FCT), and EU for labeling nascent RNA were performed or added in the sequence shown in the upper scheme as the shown black arrow line. The purple arrow line represented the period of N protein expression and the red one is for the period of nascent RNA synthesis. For immunofluorescent staining, following fixation, the cells were detected for the incorporated alkyne-modiﬁed EU with azide-derivatized Alexa 594 ﬂuorophores by using click chemistry for nascent viral RNA (red), immuno-stained with Alexa 647-mouse IgG for anti-TGEV N protein (purple). FCT was shown in green fluorescence. The resultant cells were observed using a confocal microscope for detection and **colocalization of N protein, nascent viral RNA, and fluorescent tylophorine compound**, which were merged into **white areas** in the bottom panel. They were found co-localized in the viral replication-transcription-complexes surrounding the nuclei. The fluorescence image was acquired by using a Leica TSC SP5 laser-scanning confocal microscope. Bar: 25 μm.

**Figure S3. Tylophorine treatment increased the p-p65 and p65 levels in nucleus fraction from TGEV infected ST cells.** Western analysis for p-p65 and p65 levels in nucleus and cytosol fractions from TGEV infected ST cells at the indicated m.p.i. with the indicated compound treatment. All the cells were treated with vehicle DMSO, tylophorine (1 µM), IMD-0354 (30 µM), or the combination of tylophorine and IMD-0354 for 2 h prior to TGEV infection. Results shown are representative of three independent experiments.

**Figure S4. Cytotoxicity assays for tylophorine, CYT387 and IMD-0354 in ST cells.** Crystal violet staining were performed to assess cell viability. ST cells were seeded in 96-well plates at at 8×10^4^ cells per well without or with treatments of IMD-0354, CYT387 or tylophorine as indicated at 37^o^C for 15 h. The culture medium was removed. The remained adherent cells were fixed with acetic acid/methanol (1:3, vol/vol) at room temperature for 30 min and subjected for staining with 0.5% crystal violet for 30 min. The resultant cells were then washed with tap water and dried out in chemical hood. The dried cells were added with SDS solution (1%,) to dissolved crystal violet (50 μl/well) and subjected to measure the optical absorbance at 560 nm with the Wallac Victor II system (Packard, Inc.). The mean and standard deviation were calculated. from three independent experiments each in duplicate.

**Figure S5. Association of biotinylated tylophorine with IκBα mRNA were independent of TGEV N protein. S**T cells were infected with TGEV (MOI of 7) and harvested 1 h.p.i. for preparation of total RNAs for pull-down experiments. The resultant total mRNAs (3 µg) were incubated with 30 µM of biotinylated tylophorine for 3 h at 4°C in the presence of indicated amount (ng) of recombinant N protein. The pulled down mRNA complexes were subjected to RT-PCR with the indicated specific primer pairs for IκBα or TGEV. 18/28S ribosomal RNAs were as the input loading controls for pull-down assays. Biotinylated tylophorine was labeled as Bio-Ty.


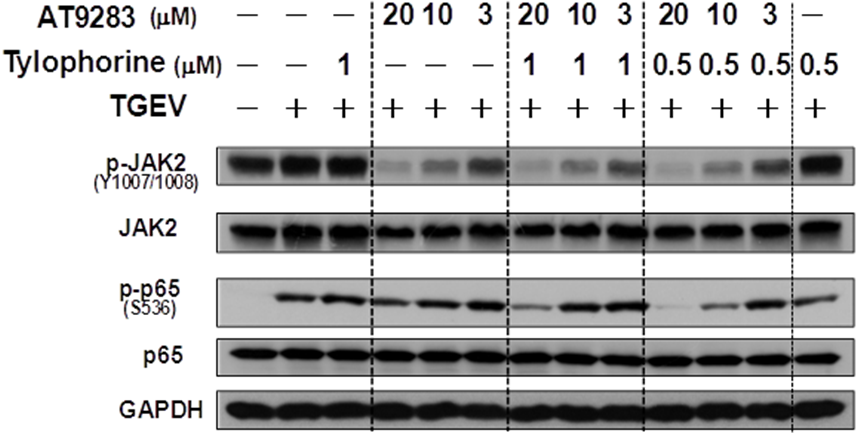


**Figure S6. Effects of JAK inhibitor AT9283 on the phosphorylation of JAK2 and p65.** All the cells were treated with vehicle DMSO, tylophorine, AT9283, or the combination of tylophorine and AT9283 1 h prior to TGEV infection (MOI of 7) and harvested at 15 min post-infection for western analysis with the indicated antibodies for p-JAK2(Y1007/1008), JAK2, p-p65 (S536), p65 and GAPDH.

**Reference.**

1. Lee, Y. Z. et al. Synthesis and biological evaluation of tylophorine-derived dibenzoquinolines as orally active agents: exploration of the role of tylophorine e ring on biological activity. J Med Chem 55, 10363-10377 (2012).
